# Health related quality of life of patients following mechanical valve replacement surgery for rheumatic mitral stenosis in Tanzania

**DOI:** 10.1186/s13019-023-02235-z

**Published:** 2023-04-21

**Authors:** Reuben K Mutagaywa, Maarten J Cramer, Pilly Chillo, Ramadhan H Khamis, Respicious Boniface, Anjela Muhozya, Aileen Barongo, Moses Byomuganyizi, Gideon Kwesigabo, Appolinary Kamuhabwa, Bashir Nyangasa, Peter Kisenge, Steven Chamuleau

**Affiliations:** 1 School of Medicine, Department of Internal Medicine, Muhimbili University of Health and Allied Sciences, P.O. BOX 5539, Dar es Salaam, Tanzania; 2 School of Medicine, Department of Surgery (Cardiothoracic& vascular section), Muhimbili University of Health and Allied Sciences, Dar es Salaam, Tanzania; 3 Muhimbili Orthopaedic Institute, Dar es Salaam, Tanzania; 4 Jakaya Kikwete Cardiac Institute, Dar es Salaam, Tanzania; 5 Division of Heart and Lung, Department of Cardiology, University Medical Centre Utrecht, Utrecht, Netherlands; 6 Department of Paediatrics, Mwananyamala Regional Referral Hospital, Dar es Salaam, Tanzania; 7 School of Public Health, Department of Epidemiology and Biostatistics, Muhimbili University of Health and Allied Sciences, Dar es Salaam, Tanzania; 8 School of Pharmacy, Department of Pharmacology and Clinical Pharmacy and Pharmacology, Muhimbili University of Health and Allied Sciences, Dar es Salaam, Tanzania; 9 Heart Center, Department of Cardiology and Amsterdam Cardiovascular Sciences, Amsterdam University Medical Centre, Amsterdam, Netherlands

**Keywords:** Rheumatic heart disease, Patient-reported outcome measures, Health related quality of life, Interventions

## Abstract

**Background:**

The assessment of outcomes of interventions based on the patient’s perspective using patient-reported outcome measures (PROMs) has been increasingly highlighted in clinical practice. However, health related quality of life (HRQoL), one of the common constructs measured by PROMs remain unknown among patients after heart valve replacement (HVR) in Tanzania.

**Objectives:**

To assess the HRQoL amongst patients operated on for rheumatic mitral stenosis at Jakaya Kikwete Cardiac Institute (JKCI).

**Methods:**

A prospective study of patients operated on due to rheumatic mitral stenosis at JKCI from January 2020 to April 2021 was undertaken. The HRQoL was assessed by using the MacNew questionnaire, addressing three domains (physical, emotional, and social function); the score ranges from 0 to 7. We categorized HRQoL as low (mean score ≤ 4.9), moderate (5–6) and high (> 6). We analysed several sociodemographic and clinical variables for HRQoL.

**Results:**

Out of 54 patients, there were 34 females and 20 males. Their mean (± SD) age was 37.98 (± 12.58) years. The reliability of translated Kiswahili version of MacNew was good. The mean (± SD) global scores were 3.47 ± 0.59, 4.88 ± 0.71 and 6.14 ± 0.50 preoperatively, at 3 months and 6 months respectively (p-values < 0.001 preoperatively vs. 3 months, preoperatively vs. 6 months and at 3 months vs. 6 months). The median of individual mean difference HRQoL score pre-operatively and at 6 months was 2.67. The preoperative and 6 months mean difference HRQoL scores were higher among patients with vs. without atrial fibrillation (2.95 ± 0.59 vs. 2.45 ± 0.53, p = 0.003) and those on anticoagulants (preoperatively) vs. not on anticoagulants (3.14 ± 0.58 vs. 2.57 ± 0.57, 0.009). The mean difference HRQoL scores were similar for sociodemographic and other clinical parameters, including those with stroke vs. without stroke.

**Conclusion:**

Six months after HVR the overall MacNew HRQoL scores improved markedly. This improvement in HRQoL was regardless of the presence of comorbidities (e.g. stroke and atrial fibrillation) which underscores the importance of considering valvular surgery if they fit the criteria. Clinicians and researchers in low-resource settings should collaborate to promote the utilization of PROMs in the routine care of patients.

## Introduction

Rheumatic heart disease (RHD) continues to be among the causes of cardiac morbidity and death among school children and young adults in Africa and the third most common cause of heart failure after hypertension and cardiomyopathy [1]. The prevalence of RHD in this region is as high as 1–3 for every 100 school children [2, 3]. Although it has been reportedly eradicated in developed countries; globalization, refugee crises and migration have led to its evolution resulting in a global health problem [4–6].

In sub-Saharan Africa, patients present late in hospital with advanced stage of RHD requiring interventions such as heart valve replacement (HVR) [7–9]. Effectiveness of surgical interventions such as HVR has been traditionally assessed based on morbidity and mortality outcomes [10, 11]. However, the impact of these interventions on the patient’s perspective assessed by patient-reported outcome measures (PROMs) is as well an important component of effectiveness as the symptomatic and functional status improvement [12, 13]. To recognize this, the American Heart Association (AHA) included health related quality of life (HRQoL) assessment as part of the treatment-impact objective for cardiac health [12]. HRQoL, one of the common constructs measured by PROMs is defined by AHA as the discrepancy between actual and desired functional status and an overall impact of health on well-being [14]. In the past two decades, PROMs have been increasingly implemented in clinical practice in countries outside of Africa [13, 15].

PROMs can be broadly divided into generic or disease-specific [16]. Generic PROMs are widely applicable and can be utilized for comparison with the general population or with patients with different characteristics or diseases. An example of generic PROMs is Short Form-36 (SF-36) [17]. Disease-specific PROMs include questions on specific health problems hence making them more sensitive to small and probably important changes in the quality of life for the patient [18]. With this advantage, disease-specific PROMs are popular among healthcare providers [16]. Example of disease-specific PROMs is the MacNew questionnaire [19, 20]. The MacNew questionnaire has been used for patients with different kinds of cardiac diseases, such as heart valve diseases, [19, 21] angina pectoris, [22] myocardial infarction, [23] heart failure, [24] and cardiomyopathy [25]. It is also used in different interventions such as heart valve surgery, [19, 21] percutaneous coronary interventions/coronary artery bypass graft, [26, 27] pacemaker implantation, [28] and cardiac rehabilitation [19, 21, 26].

With PROMs, patients are involved in the decision-making process [13, 29] and clinicians may understand how diseases and their treatment affects outcomes that are important to patients [15]. There is a scarcity of published data evaluating HRQoL outcomes for patients before and/or after HVR in developing nations [30, 31]. This study was undertaken to evaluate the HRQoL of patients in Tanzania who underwent HVR for rheumatic mitral stenosis at Jakaya Kikwete Cardiac Institute (JKCI).

## Materials and methods

### Study design and setting

This was a prospective, hospital-based cross-sectional study of Tanzanian patients who underwent mechanical HVR for moderate to severe rheumatic mitral stenosis at the cardiac surgery department at JKCI which is the only institute offering heart valve surgeries in the country. This study consecutively enrolled patients (January 2020 to April 2021) who were accepted for mechanical HVR due to moderate to severe rheumatic mitral stenosis. When mitral stenosis was associated with other valve lesions such as mitral regurgitation, aortic valve disease, and secondary tricuspid regurgitation the patients were also included. Patients with mild mitral stenosis, other forms of non-rheumatic valvular heart disease or other cardiac diseases (such as triple vessel disease necessitating coronary artery bypass graft), and biological HVR were excluded from this study. We chose to recruit patients who underwent mechanical HVR because we wanted consistent results for comparison purposes. Moreover, it the one that is most commonly done at our institute. All patients were operated on by the same local surgeons on an elective basis and all surgeries were done with the same approach.

One of our study objectives was to determine factors associated with HRQoL improvement. Based on available literature, New York Heart Association (NYHA) functional class is among those factors. From the article by Shan et al., [32] the mean (SD) HRQoL score among patients in NYHA class II and class III – IV were 5.2 ± 0.9 and 4.6 ± 0.8 respectively, p < 0.001. Using OpenEpi Software for statistical calculations [33, 34] we determined the minimum sample size to be 64. The formula used is.

1 $$n = {\left( {{Z_{\alpha /2}} + {Z_\beta }} \right)^2}*2*{\sigma ^2}/{d^2}$$

where Z_α/2_ is the critical value of the Normal distribution at α/2 (e.g. for a confidence level of 95%, α is 0.05 and the critical value is 1.96), Z_β_ is the critical value of the Normal distribution at β (e.g. for a power of 80%, β is 0.2 and the critical value is 0.84), σ^2^ is the population variance, and d is the difference you would like to detect.

### Data collection

The sociodemographic, medical and comorbidity history was obtained from all patients. New York Heart Association (NYHA) functional class score, intraoperative variables (number of valves replaced, cardiopulmonary bypass time, and aortic cross-clamp time), postoperative ICU stay, postoperative complications (congestive cardiac failure, arrhythmias, wound infection, acute respiratory distress syndrome), and mortality information was also obtained. All patients were evaluated for evidence of moderate to severe mitral stenosis according to the recommended clinical and echocardiographic parameters [35]. Several echocardiographic (e.g. pulmonary hypertension defined Right Ventricular Systolic Pressure > 35mmHg as determined by the peak systolic gradient across the tricuspid valve regurgitation [36]), electrocardiographic (e.g. atrial fibrillation) and laboratory parameters (e.g. C-reactive protein) were documented.

### Assessment of health-related quality of life

**Fig. 1:**
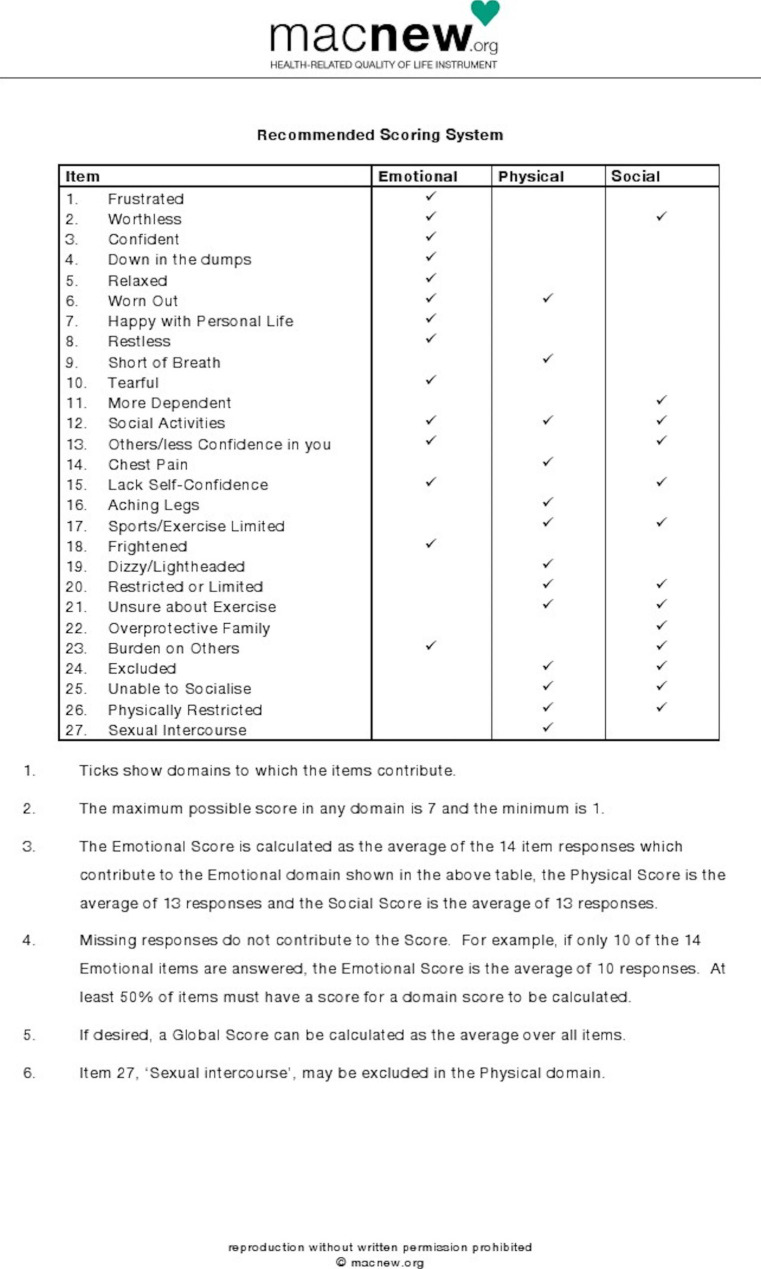
Scoring System of MacNew questionnaire

This study aimed to detect small changes in the quality of life for the patient who underwent HVR and hence the authors preferred to use the MacNew questionnaire which is an internationally accepted and valid questionnaire to assess HRQoL. It has 27 questions focusing on three important HRQoL domains namely; the physical, emotional, and social functions [19–22, 37]. The scale used is a Likert type (never = 7, very often = 6, often = 5, sometimes = 4, few times = 3, rarely = 2, always = 1). The timeframe for the MacNew is the previous two weeks. In this study, HRQOL was categorized as low (mean score ≤ 4.9), moderate (mean score between 5 and 6) and high (mean score > 6) to compare results with previous studies [38]. All the scores were transformed according to meaning. The maximum possible score in any domain is 7 (high HRQoL) and the minimum is 1 (poor HRQoL). Domain scores were calculated as the averages of the respective responses of the item. The global score was assessed as the average of all items (Fig. 1).

The questionnaire was translated into Kiswahili at the University of Dar es Salaam, Department of Kiswahili studies. A pilot study was conducted in a sample of 10 patients to ascertain the applicability of this questionnaire in the Kiswahili language and the responses were satisfactory. The internal consistency (reliability) of the Kiswahili version of the scale was good, with Cronbach’s α coefficient ranging from 0.853 to 0.911 for the subscales of Emotional, Physical, and Social domains, and 0.923 for the Global score.

We employed a pre and post-test design with completion of the questionnaires before HVR, at 3- and 6- months post-HVR. The questionnaires were interviewer-administered in which preoperatively they were administered in-person the day before HVR and postoperatively at 3 and 6 months they were either administered in-person during clinic visits or by phone for patients who could not come for follow-up visits at JKCI. To limit interview bias, the same researcher (RM) undertook all of the interviews.

### Statistical analysis

Data analysis was done using Statistical Package for Social Sciences (IBM SPSS Statistics) version 28.0 and STATA (StataCorp) version 13. Continuous data were presented as mean or median and categorical data as counts or percentages. Repeated measure ANOVA and one-way ANOVA or independent sample T-Test were used to compare different means of continuous variables. Variables which were analysed for HRQoL included patients’ age, sex, residence, education level, marital status, insurance, disease duration, NYHA class, preoperative LVEF, preoperative pulmonary arterial pressure (PAP), Tricuspid Annular Plane Systolic Excursion (TAPSE), income, preoperative atrial fibrillation (AF), left atrial size, mitral valve area (MVA), mitral valve mean pressure gradient, level of CRP, Wilkins’ grade, anticoagulants use, diuretics use, history of hypertension, history of stroke, and intraoperative variables (number of valves replaced, cardiopulmonary bypass time, and aortic cross-clamp time), and post-operative ICU stay. With each of the analyses, a p-value < 0.05 was considered statistically significant.

### Ethical consideration

We obtained written informed consent from all participants. The study was approved by the Directorate of Research and Publications of Muhimbili University of Health and Allied Sciences (P. MUHAS – REC-9-2019-059). Permission to conduct this study was obtained from JKCI (AB.157/334/01’A) ethical committee. Written permission (license: D3FCH-17E5A-2B7BB-B73EF-C1BB4-8HD5H) to utilize the MacNew questionnaire was obtained from its developers at the College of Health Sciences at the University of Wisconsin-Milwaukee.

## Results

### Patients characteristics

All patients underwent surgical intervention for RHD. The mean (± SD) age of the patients at the time of surgery was 37.98 (± 12.78) years. There were 34 females and 20 males, thus there was a female predominance (F: M = 1.7:1). The mean duration of symptoms was 6.83 ± 5.51 years. The majority, 45 (83.3%) of the patients were residing outside of Dar es Salaam. More than two-thirds of the patients were married, had primary school education and had low income while about a third had no health insurance. Twenty-four (44.4%) patients had AF, 10 (18.5%) were on anticoagulants (preoperatively), and 9 (16.7%) had previous strokes (all of them in AF and not on oral anticoagulants). The majority, 45 (83.3%) of the patients had pulmonary hypertension. About a quarter was in NYHA class III-IV and 9 (16.7%) had reduced LVEF. Nineteen (35.2%) patients had pure MS. Other lesions were 5 (9.3%) mixed mitral valve disease (MMVD), 6 (11.1%) MMVD with tricuspid regurgitation (TR), and 24 (44.4%) MMVD with aortic valve disease (AVD) and TR. The mean mitral valve area was 1.14 ± 0.39 cm^2^ and the mean trans-mitral pressure gradient was 12.54 ± 3.57 mmHg. Single (mitral) valve replacement was done in more than two-thirds of the patients (Table 1). Tricuspid valve repair was done in 16 (53.3%) patients. The type of mechanical prostheses used were Sorin Bicarbon bileaflet valves (43), Medtronic ATS mechanical heart valve (10), Bicarbon St. Jude mitral valve (1).

**Table 1:** Sociodemographic and clinical characteristics of patients on operated for rheumatic mitral stenosis at JKCI from January 2020 to April 2021 (N = 54)

Variable	Mean (±SD)/Frequency (%)
Gender (female)	34 (63)
Age of patients (years)	37.98 ± 12.78
Duration of symptoms, mean (years)	6.83 ± 5.51
Proportion residing outside of Dar Es Salaam	45 (83.3)
Proportion married	36 (66.7)
Proportion without health insurance	17 (31.5)
The proportion of low income	42 (77.8)
The proportion of primary education	35 (64.8)
Hypertensives	6 (11.1)
Patients with stroke	9 (16.7)
Patients with atrial fibrillation	24 (44.4)
Patients in heart failure NYHA class III-IV	15 (27.8)
Proportion on anticoagulants (preoperatively)	10 (18.5)
The proportion with pure MS	19 (35.2)
The proportion of pulmonary hypertension	45 (83.3)
The proportion with moderate-severe dilated LA	43 (79.6)
Mitral valve area, mean (cm^2^)	1.14 ± 0.39
Trans mitral pressure gradient, mean (mmHg)	12.54 ± 3.57
Proportion with elevated C-reactive protein	33 (61.1)
Proportion who underwent SVR	37 (68.5)

**Legend:** NYHA = New York Heart Association, MS = Mitral stenosis, LVEF =.

Left ventricular ejection fraction, TAPSE = Tricuspid Annular Plane Systolic Excursion.

LA = left atrium, SVR = Single (mitral) valve replacement.

### Duration of ICU stay, cardiopulmonary bypass, and aortic cross-clamp

The median duration of intensive care unit (ICU) stay was 2 (range, 2–3) days. The median cardiopulmonary bypass time and aortic cross-clamp time were 138 (range, 108.50–188.25) minutes and 104 (range, 76.50–144.75) minutes respectively. Two patients died within 30 days following surgery (in-hospital mortality of 3.7%).

### Clinical complications

Two (3.7%) patients died in-hospital (death within 30 days following surgery). About half of the patients experienced no complications. Non-fatal arrhythmias occurred in 8 (14.8%), two of which being AF, two bradycardias, and four sinus tachycardia. Low cardiac output syndrome and electrolyte imbalance occurred in 4 (7.4%) patients each. The frequency of occurrence of re-exploration for bleeding, acute respiratory failure, neurological dysfunction, and bleeding due to warfarin was in 2 (3.7%) patients each (Table 2).

**Table 2:** Clinical complications of patients operated on from January 2020 to April 2021 for rheumatic mitral stenosis at JKCI during the 6 months follow-up period (N = 54)

Complications	Frequency (%)
Without complications	26 (48.1)
Arrhythmias	8 (14.8)
Re-exploration for bleeding	2 (3.7)
Low cardiac output syndrome	4 (7.4)
Acute respiratory failure	2 (3.7)
Electrolyte imbalance	4 (7.4)
Sternal wound gaping	1 (1.9)
Neurological dysfunction	2 (3.7)
Bleeding due to warfarin toxicity	2 (3.7)
Warfarin resistance	1 (1.9)
Prolonged intensive care unit stay (> 5 days)	2 (3.7)

### Health-related quality of life

#### HRQoL of individual patients operated on from january 2020 to april 2021 for rheumatic mitral stenosis at JKCI (N = 50)

**Fig. 2:**
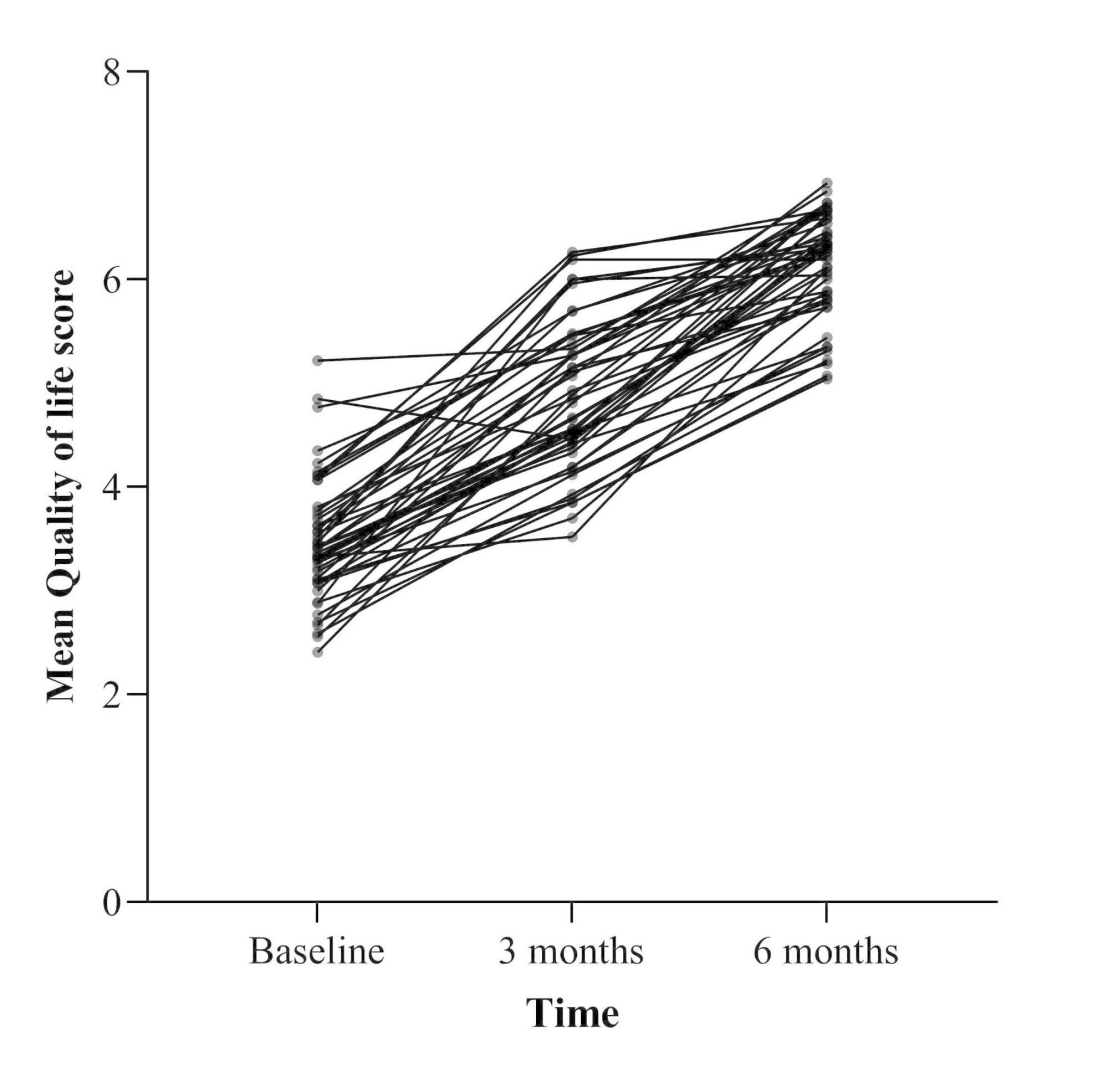
A graph showing individual HRQoL scores of patients over time

As shown in Fig. 2, the HRQoL scores per patient over time was varying from one patient to another.

#### Health-related quality of life preoperatively and over time

The preoperative MacNew questionnaire was completed by all (54) patients the day before HVR, and by 50 patients at three and six months. Preoperatively and at three months, patients had a low HRQoL (mean score ≤ 4.9). Six months after HVR the overall MacNew scores improved significantly. The mean (±SD) McNew global scores were 3.47 ± 0.59, 4.88 ± 0.71 and 6.14 ± 0.50 preoperatively, at 3 months and 6 months respectively (p-values < 0.001). Similarly, there was a significant improvement in HRQoL for the other domains (Table 3).

**Table 3:** MacNew HRQoL mean domain scores of patients operated on from January 2020 to April 2021 for rheumatic mitral stenosis at JKCI, preoperatively and over time (n = 50)

Domains	HRQoL Score			p-Value
	Preoperative Mean (±SD)	3 Months Mean (±SD)	6Months Mean (±SD)	
Global	3.47 ± 0.59	4.88 ± 0.71	6.14 ± 0.50	< 0.001
Emotional domain	3.49 ± 0.59	4.92 ± 0.77	6.16 ± 0.50	< 0.001
Physical domain	3.43 ± 0.64	4.86 ± 0.75	6.13 ± 0.58	< 0.001
Social domain	3.44 ± 0.69	4.86 ± 0.81	6.09 ± 0.58	< 0.001

**Legend:** p-value for overall trend is < 0.001.

#### Global HRQoL scores by patients’ sociodemographic characteristics

As shown in Table 4, the preoperative and 6 months mean difference HRQoL scores were similar among patients aged > 30 years and those with ≤ 30 years (2.72 ± 0.11 vs. 2.57 ± 0.60, p = 0.301) and among males and females (2.72 ± 0.66 vs. 2.64 ± 0.58, p = 0.648). Similarly, the mean difference HRQoL scores among patients with secondary vs. primary education, who are married vs. not married, having national vs. other insurance, duration of disease ≥ 10 yrs vs. < 10 yrs, and with low vs. fair income were not different. There was an improvement in the overall global mean scores, from low (mean score ≤ 4.9) preoperatively to high (mean score > 6) at 6 months in all of the analysed variable.

**Table 4:** Association of global HRQoL scores with sociodemographic characteristics of patients operated on from January 2020 to April 2021 for rheumatic mitral stenosis at JKCI, preoperatively and over time (N = 54)

Variable	Mean (± SD) Global scores		Mean difference (± SD)	*P Value
	Preoperative	6 months		
Age (yrs): ≤ 30	3.57 ± 0.65	6.14 ± 0.45	2.57 ± 0.60	0.391
>30	3.39 ± 0.57	6.14 ± 0.53	2.72 ± 0.11	
Sex: Female	3.49 ± 0.60	6.16 ± 0.46	2.64 ± 0.58	0.648
Male	3.36 ± 0.59	6.11 ± 0.57	2.72 ± 0.66	
Education: Sec - college	3.52 ± 0.55	6.11 ± 0.46	2.70 ± 0.17	0.994
Primary	3.40 ± 0.62	6.11 ± 0.53	2.68 ± 0.69	
Marital: Married	3.42 ± 0.60	6.22 ± 0.50	2.78 ± 0.59	0.152
Not married	3.50 ± 0.60	6.01 ± 0.48	2.51 ± 0.61	
Insurance: National insurance	3.44 ± 0.57	6.13 ± 0.56	2.70 ± 0.67	0.838
Public & others	3.44 ± 0.64	6.16 ± 0.41	2.54 ± 0.40	
Disease duration < 10 yrs	3.50 ± 0.57	6.14 ± 0.48	2.63 ± 0.61	0.472
≥ 10 yrs	3.32 ± 0.64	6.14 ± 0.56	2.76 ± 0.62	
Income: Low	3.40 ± 0.58	6.10 ± 0.50	2.68 ± 0.65	0.944
Fair	3.60 ± 0.65	6.26 ± 0.49	2.66 ± 0.48	

*P value for the mean difference.

#### Global HRQoL scores by patients’ clinical characteristics

Table 5 shows that the preoperative and 6 months mean difference HRQoL scores were similar among patients who were in NYHA class I-II vs. III-IV, with normal vs. low preoperative LVEF, with vs. without stroke, hypertensives vs. non-hypertensives, with vs. without pulmonary hypertension, with severe vs. moderate mitral stenosis, with elevated vs. normal CRP, and those who underwent mitral valve replacement vs. double valve. The preoperative and 6 months mean difference HRQoL scores were statistically significantly higher among patients with vs. without AF (2.95 ± 0.59 vs. 2.45 ± 0.53, p = 0.003) and those on anticoagulants (preoperatively) vs. not on anticoagulants (3.14 ± 0.58 vs. 2.57 ± 0.57, 0.009). The overall global mean scores improved from low (mean score ≤ 4.9) preoperatively to high (mean score > 6) at 6 months in all of the analysed variables.

**Table 5:** Association of global HRQoL scores with clinical characteristics of patients operated on from January 2020 to April 2021 for rheumatic mitral stenosis at JKCI, preoperatively and over time (N = 54)

Variable	Mean (± SD) Global scores		Mean difference (± SD)	*P value
	Preoperative	6 months		
NYHA class: I-II	3.43 ± 0.59	6.15 ± 0.51	2.72 ± 0.63	0.420
III-IV	3.48 ± 0.63	6.12 ± 0.50	2.56 ± 0.56	
Preop LVEF: Low	3.58 ± 0.77	6.58 ± 0.24	2.65 ± 0.72	0.930
Normal	3.42 ± 0.59	6.08 ± 0.50	2.67 ± 0.60	
Stroke: No	3.52 ± 0.56	6.12 ± 0.44	2.59 ± 0.50	0.135
Yes	3.07 ± 0.63	6.28 ± 0.49	3.20 ± 0.93	
Hypertension: No	3.49 ± 0.60	6.16 ± 0.48	2.65 ± 0.58	0.423
Yes	3.10 ± 0.30	5.89 ± 0.76	2.91 ± 0.96	
Preop PAP: Normal	3.53 ± 0.38	5.98 ± 0.38	2.47 ± 0.49	0.315
Elevated	3.42 ± 0.63	6.17 ± 0.52	2.71 ± 0.63	
Preop Atrial fibrillation: No	3.66 ± 0.61	6.14 ± 0.45	2.45 ± 0.53	0.003
Yes	3.17 ± 0.45	6.14 ± 0.56	2.95 ± 0.59	
Mitral Valve Area: Moderately reduced	3.52 ± 0.67	6.25 ± 0.47	2.66 ± 0.64	0.883
Severely reduced	3.38 ± 0.52	6.06 ± 0.54	2.68 ± 0.59	
Anticoagulants (preoperatively): No	3.53 ± 0.58	6.11 ± 0.50	2.57 ± 0.57	0.009
Yes	3.05 ± 0.51	6.27 ± 0.64	3.14 ± 0.58	
C-Reactive Protein: Normal	3.46 ± 0.63	6.06 ± 0.53	2.61 ± 0.60	0.546
Abnormal	3.42 ± 0.54	6.29 ± 0.42	2.72 ± 0.62	
Type of surgery: DVR	3.36 ± 0.60	6.05 ± 0.61	2.58 ± 0.40	0.542
SVR	3.47 ± 0.60	6.17 ± 0.46	2.70 ± 0.66	

**Legend:** NYHA = New York Heart Association, LVEF = Left ventricular ejection fraction, PAP = pulmonary arterial pressure, TAPSE = Tricuspid Annular Plane Systolic Excursion, DVR = Double (aortic and mitral) valve replacement, SVR = Single (mitral) valve replacement. *P value for the mean difference

## Discussion

### The main findings

This single-centre prospective study represents the investigation of HRQoL for patients after valve replacement for RHD in Africa and shows that preoperative HRQoL is considerably low before surgery but substantially improves post-surgery. Moreover, the improvement in HRQoL experienced post-surgery is sustained over time in all of the MacNew HRQoL domains. The preoperative and 6 months mean difference HRQoL scores were statistically significantly higher among patients with vs. without atrial fibrillation and those on anticoagulants (preoperatively) vs. not on anticoagulants. The mean difference HRQoL scores were similar for sociodemographic parameters and other clinical parameters. Patients with other comorbidities (e.g. stroke and hypertension) had a lower HRQoL at baseline but all improved after surgery to similar levels of the patients with no comorbidity. The internal consistency (reliability) of the Kiswahili version of MacNew tool was good (with Cronbach’s α coefficient ranging from 0.853 to 0.911) similar to the 0.75 to 0.89 observed in a study done by Merkouris et al. [38].

### Pre-operative HRQoL

In our study, all patients perceived that their HRQoL was impaired pre-operatively with a mean (±SD) score of 3.47 ± 0.59 on a global scale, and 3.49 ± 0.59, 3.43 ± 0.64, 3.44 ± 0.69 in the emotional, physical and social domains respectively. Our findings are similar to that reported in a study done by Mangnall et al. [10] which showed that preoperatively all mean HRQoL eight domains scores were much lower than the reference population with severe impairment in the general health and physical function domains. Similarly, Joshi et al. [39] reported that the HRQoL was impaired in all eight domains with the lowest scores in the general health and physical function domain before surgery (15.61 ± 1.30), which improved after surgery (22.95 ± 0.45). In RHD, as valve damage progresses, the consequences for the affected patients are devastating in terms of morbidity and mortality as well as poor quality of life [11, 40]. In developing countries, including sub-Saharan Africa, patients usually present in New York Heart Association (NYHA) functional class II-III, atrial fibrillation (28%), thromboembolic events (3.2%) pulmonary hypertension, and infective endocarditis [1, 7, 11, 41, 42]. These factors are signifying late presentation of patients to health facilities and/or delayed appropriate management [7, 43]. Medical treatment may retard the rate of deterioration in function, however physiological and hemodynamic changes and/or heart failure progression eventually necessitate interventions such as heart valve replacement (HVR) [7–9, 11]. Some of the patients are too late to be candidates for valvular interventions [44–46].

### Overall HRQoL

This study showed that patients who receive HVR for RHD in Tanzania experience a positive and sustained improvement in all of the MacNew HRQoL domains. There was a statistically significant improvement in the mean (±SD) MacNew global scores from 3.47 ± 0.59, 4.88 ± 0.71 and 6.14 ± 0.50 preoperatively, at 3 months and 6 months respectively. Our findings are in keeping with those reported in a study done in Nepal, South Asia which showed improvement in all domains of quality of life after valve replacement for RHD [39]. Similarly, in another study done in Fiji, an improvement was observed in all HRQoL domains except for mental health. A previous review reported a lack of published studies on HRQoL outcomes for patients with RHD pre-and/or post-heart valve surgery in developing countries [30]. Our study, together with the two cited (Fiji and Nepal) studies is addressing this scarcity. All three studies assessed HRQoL pre-operatively and post-operatively in the RHD population from resource-limited countries. However, while our study used the MacNew HRQoL questionnaire, the Nepal study assessed it by using Ferrans and Power HRQoL index, cardiac version IV and the Fiji study used the SF-36v2 questionnaire implying that the interpretation of the findings might slightly differ. Moreover, while the Nepal study translated and validated the questionnaire in the Nepalese language, our study and the Fiji study only translated the questionnaire into Kiswahili and Fijian languages respectively highlighting a need for further evaluation of the tools in future research.

### Association of global HRQoL scores with sociodemographic characteristics

#### Age

Our study showed a comparable mean difference HRQoL scores among patients aged > 30 years to those with ≤ 30 years. This could probably partly be explained by the observation that regardless of age, most of our patients had no comorbidities such as hypertension, diabetes, and infectious diseases which presumably could affect HRQoL. Our findings are similar to those reported in other studies, showing that age does not effect HRQoL after cardiac surgery [39, 47]. A study in Nepal concluded that the amount of HRQoL improvement gained by the elderly after heart valve surgery is similar to that of the young [39]. Similarly, Sedrakyan et al. [47] found that among patients who have undergone heart valve surgery, age does not limit the HRQoL advantages of surgery. Another study done among octogenarians concluded that despite a poor health status pre-operatively, an improvement in symptoms, general well-being and physical health of similar magnitude with the young counterparts was seen [48]. These observations underscore the recommendations that valve replacement should be performed even for the elderly if fits the criteria [11, 48]. However, other studies reported that younger age is associated with less HRQoL improvement [30, 49, 50]. In cardiac patients, studies reported young age as a predictor of poor HRQoL improvement [51–53]. The authors mentioned why HRQoL is better in older than younger patients: valve noise perception is likely to be exaggerated in young patients, [49] older patients have a concept that surgery has made them get better otherwise “it could be worse”, [52] and they have better psychosocial status, less anxiety and depressive behaviors [52]. Interestingly, in African culture, ageing goes along with reduced life expectancy and HRQoL and therefore elderlies probably perceive that surgery makes them feel better. Another study [10] found that older age was an independent predictor of poor HRQoL over time. Noteworthy, the observed differences between these studies could be attributed to the differences in sample size, the studied populations and the difference in the PROMs used to assess HRQoL. For example, while our study and another [39] recruited patients with RHD, other studies [47, 48] recruited patients with degenerative valve diseases.

#### Sex

In the current study, the mean difference HRQoL scores among female and male patients were similar. Our findings are in keeping with those reported in previous studies [54, 55] which have shown that post-operative HRQoL is similar among females and males. Indeed, studies have shown that sex is neither a risk factor for morbidity and mortality perioperatively nor for a prolonged intensive care unit stay [56]. Similarly, Mangnall et al. [10] have reported that male gender was a predictor of less HRQoL improvement in only one of the eight domains (the role-emotional domain) implying that there is no sex difference. Another study by Taillefer et al. [57] has shown that females had some advantage over males in terms of HRQoL improvement but the observed results on that aspect were weak. However, other studies [14, 38, 58] have demonstrated that females with cardiovascular diseases have worse HRQoL scores than males by both disease-specific and generic PROMs. These studies report that this affection is more marked preoperatively than postoperatively [55, 59] and in the physical and social domains [54–56, 59, 60]. Among other reasons, lower social supports and psychological stress among women are important determinants of poor health [61]. Given contradicting results, authors have recommended that gender perceptions and roles are important areas for research in the future [62].

### Association of global HRQoL scores with clinical characteristics

#### Atrial fibrillation/patients on anticoagulation

In this study, the proportion of patients with AF was 44.4% which is slightly higher than the approximated 30% in some studies [7, 41, 63] but lower than the 60% that was reported in another study [64]. Patients with preoperative AF had statistically significant improvement in HRQoL at 6 months when compared to those without AF. It is recognized that, even if asymptomatic, AF has a negative effect on HRQoL [65]. At presentation, patients with mitral stenosis (unlike mitral regurgitation) have advanced RHD with consequences of worse physical and emotional states [36, 66, 67]. HVR is considered to improve the well-being of these patients. Similarly, patients on anticoagulants (preoperatively) had significant improvement in HRQoL compared to those not on anti-coagulants. However, in our cohort being on anti-coagulants could be explained as a proxy for having AF. Post-operatively, all patients in this study were anticoagulated. Typically, these patients following mechanical valve prosthesis are put on anticoagulants (warfarin) with follow up International Normalized Ratio (INR) targeting therapeutic range.

#### Other clinical characteristics

Our study showed comparable HRQoL among patients who underwent double (mitral and aortic) valve replacement (DVR) with those who underwent single (mitral) valve replacement (SVR). On the contrary, Mangnall et al. [10] have reported that isolated MVR was a predictor of less improvement in HRQoL postoperatively. The authors argued that the less improvement could be due to the advanced RHD which commonly occurs with mitral stenosis (versus mitral regurgitation) at the initial presentation. However, the authors recommended further research to better understand their scenario. In our previous publication [36] we found a significantly higher mortality among patients who underwent DVR (five times) than SVR while the other study [68] reported that there was no differences between the two groups. The reported difference in mortality data could be extrapolated to HRQoL showing that the results are inconclusive and hence calling for further studies.

### Other findings

In the current study, the in-hospital mortality was 3.7% which is comparable to the 3.8% reported by Akhtar et al., [69] the 4% by Panda et al. [70] and Sharma et al., [68] 2.5% by Pillai et al., [71] and 4.4% by Debel et al. [72] This mortality rate is significantly lower than the 11% reported by Nyawawa et al. [67] and the 14% reported by Mutagaywa et al., [36] at the same centre indicating significant improvement which could probably be explained by the improved surgical techniques and perioperative care, among other reasons. Similarly, the number of complications observed in the current study is comparable with those reported by Debel et al., [72]. Moreover, in the current study, the median cardiopulmonary bypass and aortic cross-clamp time were 138 (108.50–188.25) and 104 (76.50–144.75) minutes respectively. This figure is similar (and ideal) to the mean cardiopulmonary bypass time of 137 min and aortic cross-clamp time of 102 min reported in a study done by Aditya et al. [73]. Furthermore, in this study we have found comparable HRQoL with respect to LVEF and NYHA functional class. To our knowledge, this finding has not been reported in literatures, but in our previous publications [36, 67] we found that NYHA and LVEF are not contributory to mortality. These mortality data could in a way be extrapolated to HRQoL showing that the results are not conclusive and hence highlighting a need for future research.

## Strength and limitations

This report on HRQoL among patients undergoing cardiac surgery. The study has several advantages: being a prospective design, using the disease specific questionnaire and comparing HRQoL before and after HVR. Although HRQoL is arguably correctly assessed using a qualitative approach, in the evaluation of clinical practice like in the current study, quantitative methods are the best. The small sample size of this study could not allow for the detection of the possible presence of statistical significance of association between HRQoL domains with most of the parameters. However, we analysed several parameters which revealed the association with HRQoL knowing that a lack of statistical significance does not mean that there is no clinical significance and relevance. We acknowledge that the follow up period is short, we are only reporting what happened in six months follow up and hence creating room for a prolonged follow up while increasing the sample size. Lastly, although we could not validate the MacNew tool, the translation into Kiswahili which is very well-spoken by most Tanzanians made the tool to be easily administered and accepted as is also reflected by the good internal consistency observed during a pilot study. Also, since the relationships observed in our study are comparable to that of previous studies could indicate validity.

## Conclusion and recommendations

Despite having different comorbidities, patients who receive valve surgery for RHD experience improvement in HRQoL. This study will build a body of knowledge regarding HRQoL after valve surgery for RHD in developing countries. Clinicians and researchers in low-resource settings should collaborate to promote the utilization of PROMs in the routine care of patients.

## Data Availability

The data will be available to the readers upon reasonable request.
